# Integrating environmental and neighborhood factors in MaxEnt modeling to predict species distributions: A case study of *Aedes albopictus* in southeastern Pennsylvania

**DOI:** 10.1371/journal.pone.0223821

**Published:** 2019-10-17

**Authors:** Daniel Wiese, Ananias A. Escalante, Heather Murphy, Kevin A. Henry, Victor Hugo Gutierrez-Velez

**Affiliations:** 1 Department of Geography and Urban Studies, College of Liberal Arts, Temple University, Philadelphia, Pennsylvania, United States of America; 2 Department of Biostatistics and Epidemiology, College of Public Health, Temple University, Philadelphia, Pennsylvania, United States of America; 3 Department of Biology, College of Science and Technology, Temple University, Philadelphia, Pennsylvania, United States of America; Universidad de Sevilla, SPAIN

## Abstract

*Aedes albopictus* is a viable vector for several infectious diseases such as Zika, West Nile, Dengue viruses and others. Originating from Asia, this invasive species is rapidly expanding into North American temperate areas and urbanized places causing major concerns for public health. Previous analyses show that warm temperatures and high humidity during the mosquito season are ideal conditions for *A*. *albopictus* development, while its distribution is correlated with population density. To better understand *A*. *albopictus* expansion into urban places it is important to consider the role of both environmental and neighborhood factors. The present study aims to assess the relative importance of both environmental variables and neighborhood factors in the prediction of *A*. *albopictus*’ presence in Southeast Pennsylvania using MaxEnt (version 3.4.1) machine-learning algorithm. Three models are developed that include: (1) exclusively environmental variables, (2) exclusively neighborhood factors, and (3) a combination of environmental variables and neighborhood factors. Outcomes from the three models are compared in terms of variable importance, accuracy, and the spatial distribution of predicted *A*. *albopictus’* presence. All three models predicted the presence of *A*. *albopictus* in urban centers, however, each to a different spatial extent. The combined model resulted in the highest accuracy (74.7%) compared to the model with only environmental variables (73.5%) and to the model with only neighborhood factors (72.1%) separately. Although the combined model does not essentially increase the accuracy in the prediction, the spatial patterns of mosquito distribution are different when compared to environmental or neighborhood factors alone. Environmental variables help to explain conditions associated with mosquitoes in suburban/rural areas, while neighborhood factors summarize the local conditions that can also impact mosquito habitats in predominantly urban places. Overall, the present study shows that MaxEnt is suitable for integrating neighborhood factors associated with mosquito presence that can complement and improve species distribution modeling.

## Introduction

Climate change, particularly affected by anthropogenic processes, are likely to exacerbate the expansion of *Aedes albopictus* (Skuse) into higher latitudes [1]. Although *A*. *albopictus* is typically regarded as a rural vector [2–4] this mosquito is becoming pervasive in urban and suburban environments, where it finds suitable environments to reproduce such as artificial containers (e.g. discarded tires and trash) and built-environment features (e.g., storm water structures) as well as humans, their preferred blood source [5–8]. In addition, the urban heat island effect [9] along with land cover changes connected to urbanization are known to increase mosquitoes presence in populated areas [10]. Increasing urban population growth in along with an increasing presence of *A*. *albopictus*, constitute a public health concern given the risk of transmission of vector borne diseases.

Mosquitoes belonging to the *Aedes* genus are responsible for the transmission of several infectious diseases. After *A*. *aegypti*, the “Asian Tiger Mosquito”–*A*. *albopictus* is one of the main vectors of the Zika (ZIKV), West Nile (WNV), Dengue (DV) and Chinkunguya (CHIKV) viruses [11–13]. Despite the tropical origin of this invasive species [14], the North American *A*. *albopictus* strain most likely originates from northern Asia [15], and is rapidly expanding into temperate areas of North America. This expansion is explained in part by the capacity of this species to reproduce in areas with colder and more seasonal temperatures [16]. Its presence appear to be predicted by the average relative humidity in human inhabited areas. Furthermore, *A*. *albopictus* eggs undergo diapause allowing it to resist low temperatures and drought [17]. Previous studies show that optimal environmental conditions, such as mild winter temperatures (0° to -5°C) [18], absence of long dry periods [19], high humidity during the mosquito season, as well as vegetation are important factors that constitute a suitable habitat for *A*. *albopictus* [20] and can extend their lifespan [21]. *A*. *albopictus* distribution is also directly correlated to population density and land-cover class [14, 22, 23].

Neighborhood factors are often used in health and environmental research to summarize the economic, social, and physical conditions of places or to account for differences in social context and local policies [24]. Neighborhood socioeconomic factors (e.g. poverty) and neighborhood conditions such as land cover fragmentation and landscape heterogeneity have been shown to be important predictors of suitable mosquito habitats [25, 26]. Several studies found that high poverty neighborhoods have a higher probability of building abandonment, dumping and more trash and containers that are conducive to breeding *A*. *albopictus* [22, 27]. Considering neighborhood factors along with environmental variables is particularly important because it provides a neighborhood based socio-ecological approach to understanding the abundance and composition of mosquitos in different habitats.

Machine-learning methods have gained popularity for species-distribution modeling in recent years [28]. Generally, two types of algorithms are used: those that predict species distribution using both presence and absence data, and those based on species presence only. For example, among models that require only presence data, MaxEnt (Maximum Entropy) algorithm has become popular for modeling species distribution [29] since its release in 2006 by Phillips et al. [30]. Popularity of this machine-learning method is related to its ability to incorporate background data, accounting for environmental variations across space [31]. Additionally, machine-learning algorithms like MaxEnt are efficient modeling approaches because of their ability to fit highly complex responses [32].

MaxEnt [30] has mostly been used to predict the distribution of *A*. *albopictus* in Northeastern United States using environmental data [1]. Only a few studies that focused on *A*. *albopictus*, have incorporated neighborhood socioeconomic determinants (also neighborhood factors; e.g. conditions of neighborhoods or area-based socioeconomic measures) [22, 25, 27], and to our knowledge, no studies on species-distribution modeling have incorporated neighborhood factors using MaxEnt. Sallam et al [22] argue that based on a literature review on *Aedes* genus habitat modeling, MaxEnt appears to be the most appropriate tool, whereby land cover, meteorological, neighborhood socioeconomic determinants must be considered.

This study aims to assess the relative importance of both environmental variables and neighborhood factors in the prediction of *A*. *albopictus*’ presence in SE Pennsylvania. This work demonstrates the utility of the MaxEnt method beyond typical applications using only environmental variables by also including neighborhood factors. The combination of environmental and neighborhood variables in the model can inform management agencies about the relative influence of environmental and social drivers of the spatial distribution of mosquitos in urban environments.

## Materials and methods

### Ethics statement

The collection of mosquitoes did not require permissions because they were conducted with homeowners consent and by county mosquito control professionals. The study did not involve any endangered or protected species.

### Study area

SE Pennsylvania is located within a highly urbanized corridor extending between Washington D.C. and New York City, NY. Therefore, the selected study area, dominated by built environments, and a high population density is an ideal region for exploring the influence of natural environmental factors as well as neighborhood conditions on the presence of *A*. *albopictus*.

**Fig 1:**
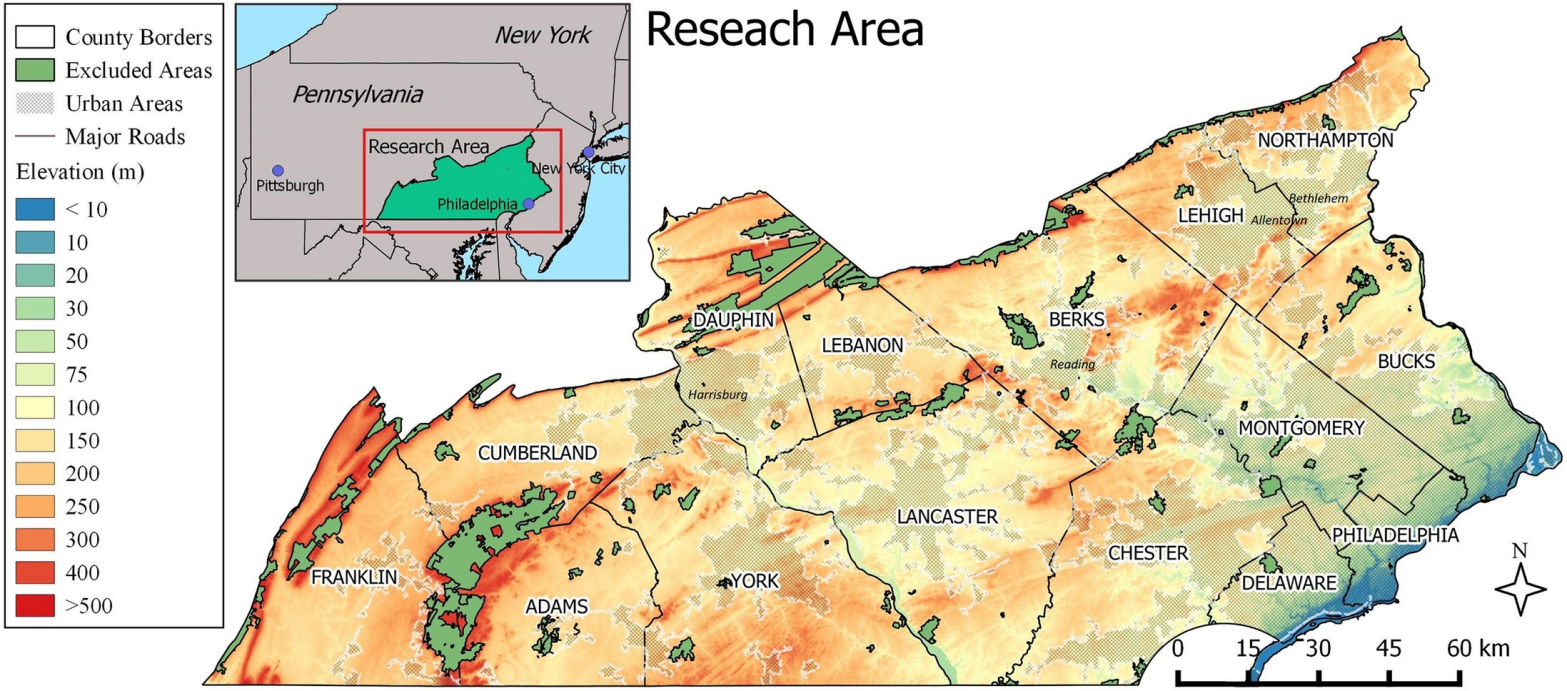
Research Area in Southeast Pennsylvania. Urban areas are shown as defined by the US Census Bureau.

The study area includes 15 counties in SE Pennsylvania (Fig 1). The region is bounded by the Delaware River and the state of New Jersey to the East; the Blue Ridge and Ridge-and-Valley regions to the West, Northwest and the North; the Delaware and Maryland state borders to the South. The study area is 21,874 km^2^. According to the 2010 United States Census, the total population of the region is 6,978,996. The most populated areas, Philadelphia County and its suburban surrounded areas, can be found in the Southeast of the state. Additional large settlements are Bethlehem and Allentown in the North, Reading in the North-Central, and Lancaster, York and Harrisburg in the Central parts of the study area. In total, the urban/urbanized regions cover 31% of the study area [33].

The climate of the study area is characterized by a cold winters, hot summers, a non-marked dry season [34], and almost equally distributed precipitation throughout the entire year [35]. The study area has low inter-annual climate variability. The differences in temperature and precipitation are related mostly to altitude [35]. The average annual precipitation is approximately 1200mm (driest month: February, ca. 81mm; wettest month: July, ca. 120mm). The average temperature is approximately 10.8°C (7.9°C-13.8°C). A previous study by Rochlin et al [1] showed that expected changes in climate will affect the distribution of *A*. *albopictus* in the Mid-Atlantic region of the U.S., resulting in the expansion of these species to northern latitudes.

### Data

Mosquito data included 129,476 records from single mosquito traps installed during the months March through November, between 2001 and 2015. *Aedes albopictus* was captured in 38,515 traps at 8,801 unique locations. The majority of traps were gravid traps and miniature light traps from the Centers for Disease Control and Prevention (CDC). Other methods such as aspiration, mosquito magnets, BG Sentinel traps and Zumba traps were sometimes used and varied by year. The Division of Vector Management within the Pennsylvania Department of Environmental Protection (DEP) installed and monitored the traps. The publically available data were provided by the DEP (processed data are attached, for original data please contact mhutchinso@pa.gov or RA-epcontactus@pa.gov), and included information about location of the trap, time of installation and the number of captured female *A*. *albopictus* adults. In urban areas, most traps were installed in close proximity to storm water sewers. While in rural areas, traps were placed mostly along major routes of transportation, water canals and reservoirs but were not placed in any of the State Parks, State Forests and Game Lands or on private facilities nor those managed by military or any other authority.

The independent variables were grouped as neighborhood factors and environmental site factors (Tables 1 and 2). Neighborhood factors included Census data collected at the ZIP Code Tabulation Area (ZCTA) level as well as National Land Cover Database (NLCD 2011) rasters. Census data were obtained from the National Historic GIS [33], and included the data from decennial 2000 and 2010 United States Censuses, as well as the American Community Survey (ACS) 5-year-average estimates from 2007–2011 and 2010–2014.

**Table 1:** List of collected neighborhood factors.

Variable Name	Description	Original Format	Selection Criteria	Calculation	Source
Below Poverty	Area-based percent population below poverty line	csv file, 5-years-average estimates 2007–2011 and 2010–2014	Recommended by Sallam et al [22]	$\frac{Population\;below\;poverty\;}{Total\;Population}$	ACS
Best Housing Conditions	Area-based percent of housing units with no selected physical or financial conditions	csv file, 5-years-average estimates 2007–2011 and 2010–2014	Own selection. Not correlated to any other variable.	$\frac{Houses\;without\;conditions\;}{Total\;houses\;}$	ACS
Education	Area-based education index	csv file, 5-years-average estimates 2007–2011 and 2010–2014	Recommended by Rochlin et al [25].	∑i=1n(Peoplei∑i=1nPeoplei) *i* = class number, *n* = number of classes	ACS
Median Household Income	Area-based median household income in USD	csv file, 5-years-average estimates 2007–2011 and 2010–2014	Recommended by Rochlin et al [25].	Original value in data source	ACS
Housing Density	Housing density per square kilometer	csv file, 5-years-average estimates 2007–2011 and 2010–2014	Recommended by Sallam et al [22].	$\frac{Housing\;units}{Extent\;of\;the\;ZCTA\;}$	ACS
Population Density	Population density per square kilometer	csv file, 5-years-average estimates 2007–2011 and 2010–2014, Decennial Census 2000, 2010	Excluded. Strong correlation with housing density.	$\frac{Population}{Extent\;of\;the\;ZCTA\;}$	ACS, US Census Bureau
Urban Population	Area-based percent urban population	csv file, Decennial Census 2000, 2010	Excluded. Strong correlation with population density.	Original value in data source	US Census Bureau
Vacant Housing Units	Area-based percent vacant housing units	csv file, 5-years-average estimates 2007–2011 and 2010–2014	Recommended by Rochlin et al [25].	$\frac{Vacant\;housing\;units}{Total\;housing\;units}$	ACS
Worst Housing Conditions	Area-based percent of housing units with four selected physical or financial conditions	csv file, 5-years-average estimates 2007–2011 and 2010–2014	Own selection. Not correlated to any other variable.	$\frac{Houses\;with\;4\;conditions\;}{Total\;houses\;}$	ACS
Imperviousness of the surfaces	Percent impervious surfaces	Raster, 30m resolution, 2011	Recommended by Sallam et al [22].	Original value in data source	NLCD
Land Cover	Type of Land Cover Class	Raster, 30m resolution, 2011	Recommended by Sallam et al [22].	Original value in data source	NLCD

**Table 2:** List of all collected environmental variables.

Variable Name	Description	Original Format	Selection Criteria	Calculation	Source
Tree Canopy	Percent of tree canopy per pixel	Raster, 30m resolution, 2011	Own selection. Not correlated to any other variable.	Original value in data source, resampled to 232m spatial resolution	NLCD
Average Precipitation in November, December, January, February, March, April, May, June, July, August, September, October (mm)	Average precipitation for each month for the years 2000–2015	180 raster files, 4km resolution, monthly values for Oct. 2000 –Nov. 2015	Rochlin et al [1] found strong association with Average January Precipitation	Original value in data source, resampled to 232m spatial resolution	PRISM
3-Month Average Precipitation starting November, December, January, February, March, April, May, June, July, August, September, October (mm)	Average precipitation for each 3-months for the years 2000–2015	180 raster files, 4km resolution, monthly values for Oct. 2000 –Nov. 2015	Rochlin et al [1] found strong association with Wettest and Driest Quarter Precipitation	Original value in data source, resampled to 232m spatial resolution	PRISM
Average Temperature in November, December, January, February, March, April, May, June, July, August, September, October (°C)	Average temperature for each month for the years 2000–2015	180 raster files, 4km resolution, monthly values for Oct. 2000 –Nov. 2015	All variables are highly correlated with quarter year temperatures	Original value in data source, resampled to 232m spatial resolution	PRISM
3-Month Average Temperature starting November, December, January, February, March, April, May, June, July, August, September, October (°C)	Average temperature for each 3-months for the years 2000–2015	180 raster files, 4km resolution, monthly values for Oct. 2000 –Nov. 2015	Rochlin et al [1] found strong association with Coldest Quarter Temperature	Original value in data source, resampled to 232m spatial resolution	PRISM
Average EVI	Average EVI value of the mosquito season for the study period	Raster, 232m resolution, monthly values for Apr-Oct 2001–2015	Own selection. Not correlated to any other variable.	Original value in data source	MODIS
Average NDWI	Average NDWI value of the mosquito season for the study period	Raster, 232m resolution, monthly values for Apr-Oct 2001–2015	Own selection. Not correlated to any other variable.	Original value in data source	MODIS
Elevation (DEM)	Elevation in meters above the sea level	Raster, 90m resolution	Own selection. Not correlated to any other variable.	Original value in data source, resampled to 232m spatial resolution	SRTM
Slope	Slope in degrees	Raster, 90m resolution	Own selection. Not correlated to any other variable.	Calculation based on the DEM using ArcGIS 10.5 tool Slope, resampled to 232m spatial resolution	SRTM
Flow Accumulation	Flow accumulation index	Raster, 90m resolution	Own selection. Not correlated to any other variable.	Calculation based on the DEM raster using ArcGIS 10.5 tools Flow Direction and Flow Accumulation, resampled to 232m spatial resolution	SRTM

Neighborhood measures were included because previous studies by Rochlin et al [25], Little et al [27] indicated the importance of education level, percent of vacant housing and household income as predictors of mosquito presence. Others noted the importance of median income and vacant lots in predicting mosquito presence [22]. Additionally, we decided to examine the effects of housing conditions, housing density, poverty level as well as population density and proportions of urban population per ZCTA (Table 1). The NLCD 2011 was used as the source for the Landsat-based raster land cover (LC) inputs, as well as the data summarizing the percentage of imperviousness and tree canopy cover. We decided to classify the LC and imperviousness rasters as neighborhood factors because of the dominance of built environments in the study area that shape the character of many urban neighborhoods. A previous study by Rochlin et al [25] also showed that land cover type and especially, natural habitat fragmentation caused by road networks affect mosquito presence.

Environmental variables (Table 2) included: spectral indices (Enhanced Vegetation Index (EVI), and Normalized Difference Water Index (NDWI), terrain parameters, precipitation and temperature. EVI and NDWI were obtained from data acquired by the MODIS instrument aboard NASA’s terra satellites (MOD013Q1 and MOD09Q1 data products). Terrain parameters included: elevation, slope and flow accumulation. Slope and flow accumulation were extracted from a Digital Elevation Model derived from the Shuttle Radar Thematic Mapper (SRTM) satellite using tools “Slope” and “Flow Accumulation” in ArcGIS version 10.5. Precipitation and temperature were derived from the Parameter-Elevation Regressions on Independent Slopes (PRISM) analytical climate model. PRISM was developed through the interpolation of point data from climate stations, considering factors such as: location, elevation, coastal proximity, topographic facet orientation, vertical atmospheric layer, topographic position, and orographic effectiveness of the terrain [36, 37].

Additionally, geographic files (e.g. GIS shapefiles) of Pennsylvania State Parks, State Forests, Game Lands and other environmental protected areas from the Pennsylvania Spatial Data Access website (http://www.pasda.psu.edu/) were used to generate a mask to exclude large bodies of areas unrepresented in the sampling.

### Analysis

**Fig 2:**
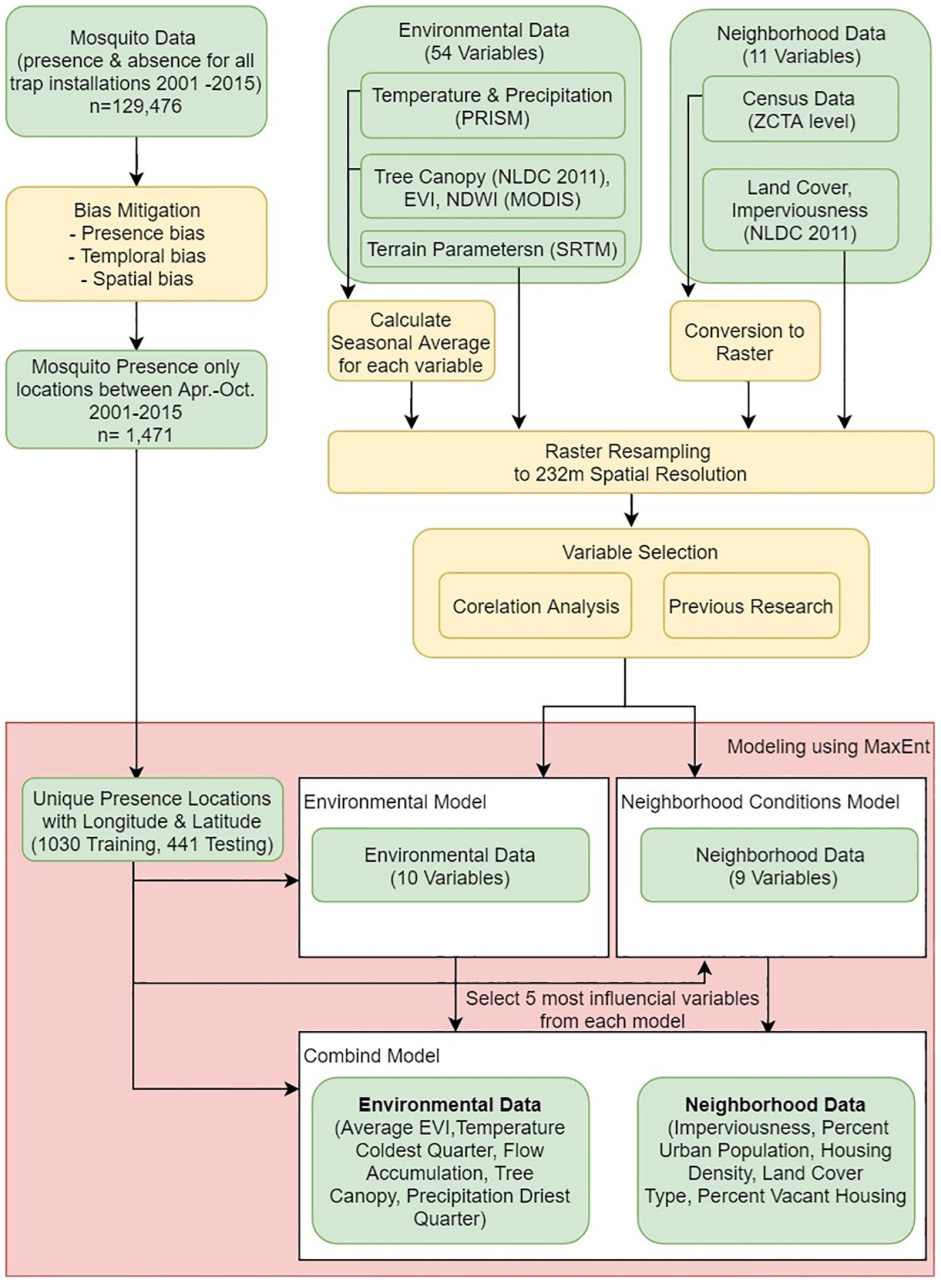
Methodological workflow.

The methodological workflow followed several steps including: data preparation and preprocessing to meet MaxEnt (version 3.4.1) requirements, correlation analysis in order to exclude collinear variables, a three-step modeling process, and then visualization of the results. All stages of the workflow are shown in Fig 2, and described in the following sections.

#### Preprocessing of mosquito data

**Fig 3:**
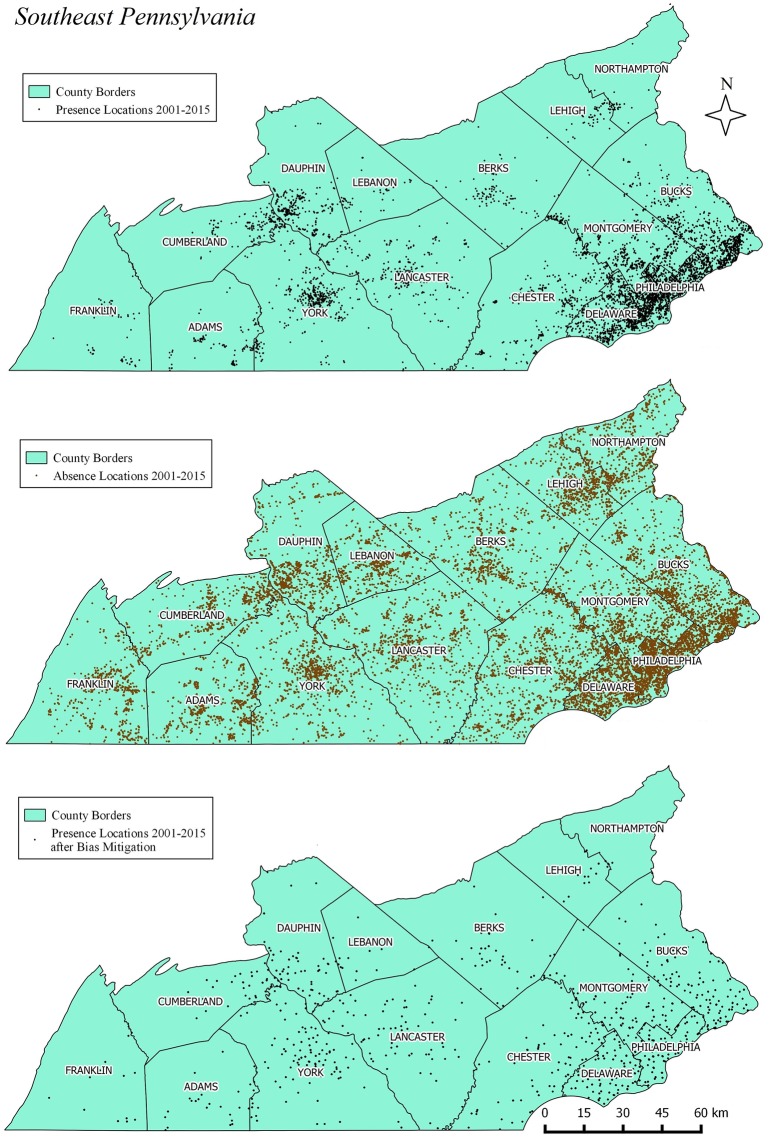
Location of Mosquito Traps 2001–2015. Upper map shows all original presence locations; middle map shows all absence location; bottom map shows remaining presence location after bias mitigation.

MaxEnt predicts species distributions based on presence data only [31]. The algorithm requires a list of geographic coordinates representing species presence and the values of the predicting environmental raster variables associated with those coordinate locations. Although the mosquito database contains absence data, most of the locations that resulted in absences were measured only a few times (34% of the absence locations were sampled only once while 62% of them were sampled four times or less). Given the high annual seasonality of mosquito populations and the low sampling frequency of the absence observations, the inclusion of such imperfect data in the model can introduce estimation biases and also inflate accuracy estimates [38]. Therefore, the use of MaxEnt with only presence observations was deemed appropriate for the current dataset. A geographic filter was applied to mitigate the evident sampling location bias in the study area (Fig 3). This method has been proposed as an approach to reduce the effect of sampling bias on model overfitting [39, 40], and performs better than other methods for reduction of sampling location bias [41]. Geographic filtering was applied by dividing the study area in 10x10 MODIS pixel resolution cells and then selecting one sampled location per grid cell randomly to run the model. Finally, the coordinates of the remaining mosquito-presence locations (n = 1,471) were exported as a CSV file (S1 Table) to meet MaxEnt import requirements.

#### Preprocessing of Census data

The US Census Bureau publicly releases the results of the decennial census as well as the estimates from the ACS 5-year-average. For our purpose, the original format (count data) was converted into density or percentage to ease the comparison of data between areas in the study region. Then, every calculated area-based socioeconomic measure was joined to the ZCTA shapefile and converted into raster format using R package raster version 2.5–8 [42]. For all available years of each Census variable, we then calculated an average value.

#### Preprocessing of remote sensing data

All environmental variables (Table 2) were obtained in raster format but with different resolution and projection. Therefore, we aligned all the raster input data to the spatial resolution and projection of the MOD013Q1 product (~250m sinusoidal). The selected resolution was a good compromise between the different spatial resolutions of the input data and was in agreement with the reported flying distance of *A*. *albopictus* species of about 250 meters [43]. All resampling procedures were performed using the resampling function within the raster package in R 3.3.1 software [42].

Monthly precipitation and temperature data were obtained for the period of November 2000 to October 2015 and were summarized to monthly average rasters. Three-month moving averages starting the November prior to the mosquito season were also calculated.

The original NLCD 2011 rasters for imperviousness and canopy cover as well as the PRISM rasters were resampled using a bilinear interpolation, while the resampling of the categorical LC raster was performed by assigning the most dominant class of the original pixels to each new resampled grid cell using the R package raster [42]. While all NLCD 2011 products were resampled from 30m to 250m resolution, PRISM climate rasters were rescaled from 4km to 250m spatial resolution.

#### Variable selection

The database included 65 explanatory variables. Fifty-four variables represented climatic conditions, three variables represented terrain parameters, and three characterized vegetation condition, while the remaining 11 variables were considered as neighborhood factors (Tables 1 and 2). To avoid collinearity issues, model overload, and to ease the comparison of the final results, the number of independent variables for each model were restricted to a maximum of 10. To make the final selection, a two-step exclusion process was performed. First, a Pearson correlation analysis between all 65 variables using R [44] was applied, and highly correlated variables were excluded (r^2^>0.5). Priority was given to variables that were significantly influential in previous studies in the Mid-Atlantic region of the U.S. [1, 25, 26]. The final database included 10 environmental variables (Model 1) and 9 neighborhood factors (Model 2). For the third, combined model, up to five variables were selected based on their highest percent contribution in models 1 and 2 and their permutation importance being at ca. 5% (Fig 2).

#### Modeling using MaxEnt

Three predictive models were generated for *A*. *albopictus* presence utilizing environmental and neighborhood conditions variables as well as coordinates of mosquito presence (n = 1,471) recorded between 2001 and 2015. For each of the three models, identical settings were applied including number of iterations (12,000) and logarithmic format of output. Thirty percent of the presence data (n = 441) were used for validation tests. Since the reported flying distance of *A*. *albopictus* species is about 250 meters [43], an option of adjusted sample radius of 250 m was applied. All selected MaxEnt parameters are also displayed in S2 Table.

#### Postprocessing

The outputs of the three models were used to generate maps representing the habitat suitability for *A*. *albopictus* in the range from 0 to 1. Pixels were then reclassified as presence/absence maps in QGIS version 2.18.7. As the threshold, MaxEnt’s *maximum training sensitivity plus specificity occurrence* method was applied, which has been identified as the most promising threshold in the prediction of presence data only [45]. Model accuracy was measured through the Area Under Curve (AUC) for the test data of the Receiver Operating Characteristic or ROC [46], which is internally calculated in MaxEnt, and is a reliable criteria for model selection [47].

## Results

### Variable importance

**Fig 4:**
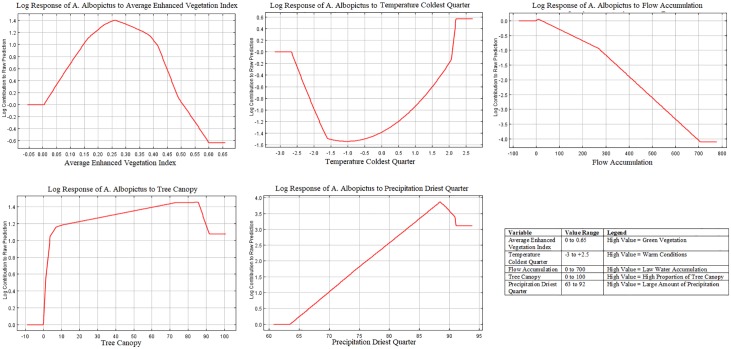
Response Curves of *A*. *albopictus* to most important variables in Model 1 (environmental variables only).

The results for model 1 (environmental variables only) show that the most influential variables predicting *A*. *albopictus* were: Enhanced Vegetation Index, EVI (28.8% contribution), average temperature of the coldest quarter (December-February) (25.1% contribution), flow accumulation (high value indicates areas with large water flow, such as channels) (13.4% contribution), tree canopy (11.1% contribution) and precipitation of the driest quarter (January-March) (5.9% contribution). Moreover, each of these variables had a permutation value higher that 5% indicating that model performance would drop without each of these variables (Table 3). Model 1 with environmental variables predicted *A*. *albopictus*’ presence in areas associated with high proportions of tree canopy and average EVI of 0.25 (overall sparsely vegetated land), and a high degree of water flow accumulation (e.g. channels, gullies). Warm temperatures during the coldest quarter (December-February) and the increasing amount of precipitation up to 87mm during driest quarter (January-March) are important as well (Fig 4).

**Table 3:** Comparison of the three models in accuracy, variable importance and contribution.

Model	Accuracy AUC test	Variables	Percent Contribution	Percent Permutation	Sensitivity	Specificity
**Model 1: Environmental Variables Only**	73.5%	**Average EVI**^1^	**28.8**	**19.2**	71.5%	71.5%
		**Temperature Coldest Quarter**	**25.1**	**5.3**		
		**Flow Accumulation**	**13.4**	**7.7**		
		**Tree Canopy**	**11.1**	**7.4**		
		**Precipitation Driest Quarter**	**5.9**	**34.6**		
		Slope	5.7	2.2		
		January Precipitation	3.7	16.4		
		Precipitation Wettest Quarter	3.4	5.4		
		NDWI^2^	2.3	1.5		
		Elevation	0.6	0.5		
**Model 2: Neighborhood Factors Only**	72.1%	**Imperviousness**	**59.0**	**64.9**	68.2%	55.3%
		**Percent Urban Population**	**19.2**	**7.1**		
		**Land Cover Type**	**7.1**	**6.1**		
		**Housing Density**	**6.2**	**13.4**		
		**Percent Vacant Housing**	**2.6**	**4.8**		
		Education Level	2.5	1.1		
		Percent Below Poverty	2.5	1.0		
		Best Housing Conditions	0.7	1.4		
		Worst Housing Conditions	0.1	0.2		
**Model 3: Final Model with Environmental and Neighborhood Factors**	74.7%	Imperviousness	42.6	29.7	72.1%	67.8%
		Percent Urban Population	16.0	8.5		
		Average EVI^1^	10.4	12.2		
		Temperature Coldest Quarter	10.0	20.9		
		Tree Canopy	6.2	9.7		
		Precipitation Driest Quarter	5.7	3.0		
		Land Cover Type	4.3	6.2		
		Percent Vacant Housing	2.9	5.1		
		Flow Accumulation	1.5	3.8		
		Housing Density	0.2	1.1		

Note: Variables with contribution or permutation at ca. 5% are marked bold.

^1^EVI- Enhanced Vegetation Index,

^2^NDWI- Normalized Difference Water Index

**Fig 5:**
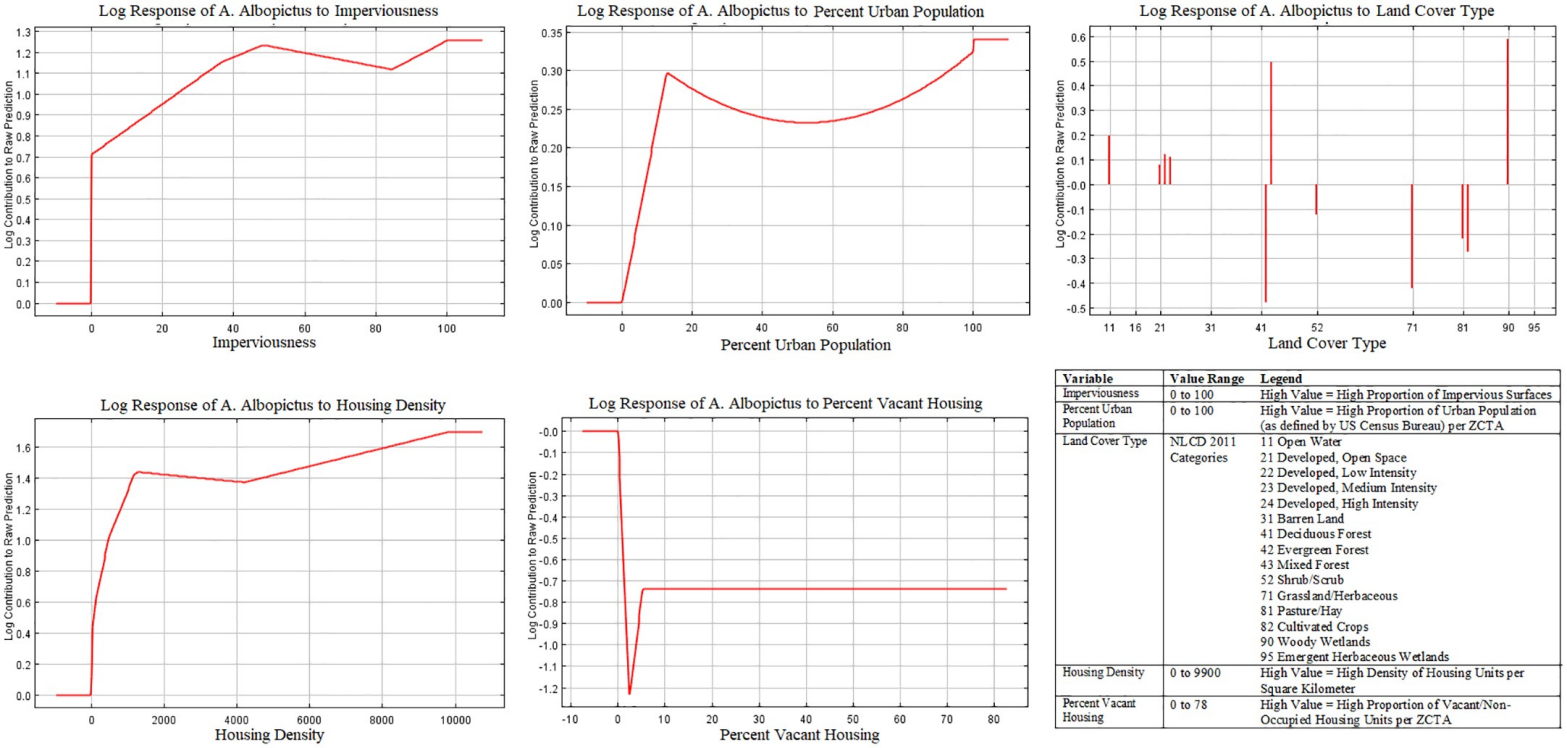
Response Curves of *A*. *albopictus* to most important variables in Model 2 (neighborhood factors only).

The most influential variables for model 2 with neighborhood factors were: imperviousness (59.0% contribution, 64.9% permutation importance), percent urban population (19.2% contribution, 7.1% permutation importance), land cover type (7.1% contribution, 6.1% permutation importance), housing density (6.2% contribution, 13.4% permutation importance), percent vacant housing (2.6% contribution, 4.8% permutation importance). Therefore, this model predicted mosquito presence predominantly in areas with a high proportion of impervious surfaces, large urban population and high-density housing. While the proportion of vacant housing appeared to be an influential component based on the permutation importance (Table 3), its influence on *A*. *albopictus’* presence remained constant (Fig 5) and showed a negative relationship. Additionally, various land cover classes had different associations with *A*. *albopictus’* presence (Fig 5). While woody wetlands, mixed forest, open water and open, low or medium intensity developed lands were positively associated with *A*. *albopictus*’ presence, other classes such as evergreen forest, shrubs, grasslands, pastures or croplands showed a negative relationship (Fig 5).

**Fig 6:**
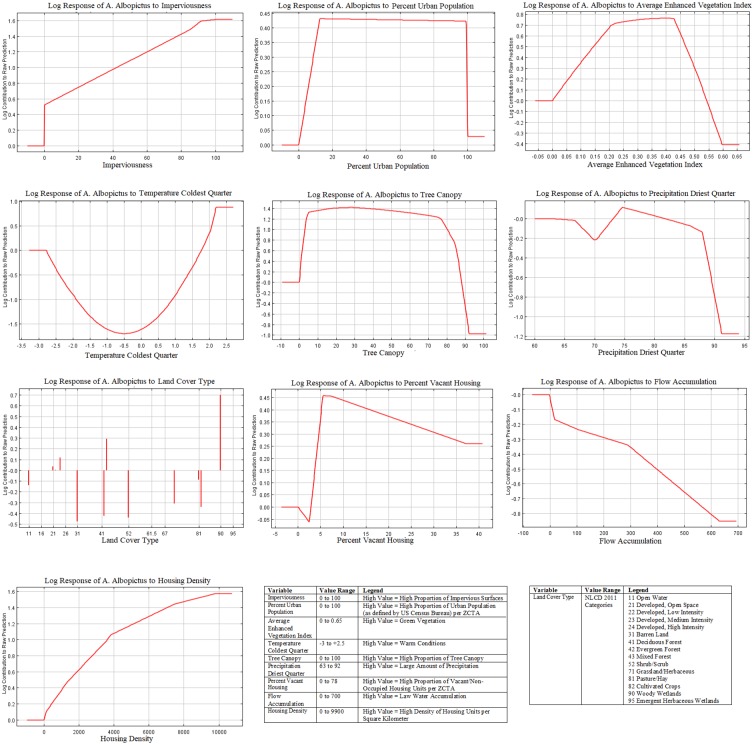
Response Curves of *A*. *albopictus* for all variables in Model 3 (environmental and neighborhood factors).

The most important variables for model 3 with environmental and neighborhood factors with contribution and or permutation importance higher than 5% were: imperviousness (42.6% contribution, 29.7% permutation importance), percent urban population (16% contribution, 8.5% permutation importance), average EVI (10.4% contribution, 12.2% permutation importance), temperature of the coldest quarter (10% contribution, 20.9% permutation importance), tree canopy (6.2% contribution, 9.7% permutation importance), precipitation of the driest quarter (5.7% contribution, 3% permutation importance), land cover type (4.3% contribution, 6.2% permutation importance), and percent vacant housing (2.9% contribution, 5.1% permutation importance) (Table 3). The combined model showed that a higher chance of *A*. *albopictus’* presence was related to increasing proportion of impervious surfaces and an urban population higher than 12%. The most suitable average EVI for mosquito presence was between 0.2 and 0.43 (sparsely vegetated to medium-dense vegetated land) as well as tree cover of 7–78%. Mild temperatures during the coldest period (December to February) were also related to a higher presence of *A*. *albopictus* (Fig 6). The response of *A*. *albopictus’* presence prediction and precipitation during the driest quarter had negative association, and was inconstant. Optimum precipitation is achieved at 74mm and a rapid decline in mosquito presence when precipitation is higher than 88mm. In contrast to model 2, land cover class showed a slightly different relationship. Open water was now negatively associated with *A*. *albopictu*s’ presence, while high intensity developed land was positively associated with *A*. *albopictu*s’. The response curve for percent of vacant housing also changed in comparison to model 2, indicating that the most suitable conditions for mosquito proliferation were in areas with 5–8% vacant housing units (Fig 6). Finally, flow accumulation and housing density were the least important variables with the lowest contribution and permutation values (Table 3), while their association with *A*. *albopictus’* presence remained similar to the results from model 1 and model 2. The high level of flow accumulation as well as increasing housing density had positive associations (Fig 6).

### Models’ accuracy

According to the ROC for model 1(environmental variables), the AUC value on test data was about 0.735, or an overall accuracy of 73.5%. Model 2 (neighborhood factors) had an overall accuracy of 72.1%, while the combined model resulted in an accuracy of 74.7%. In model 3 (environmental and neighborhood factors) did not substantially increase the overall accuracy of the model based on the AUC test data results.

Comparing sensitivity (true-positive/true-positive+false-negative) and specificity (true-negative/true-negative + false-positive) rates, the model 2 (neighborhood factors) had the lowest rates (68.2% and 55.3% respectively), while environmental factors model 1 achieves a balanced ratio for sensitivity and specificity both at 71.5%. Model 3 (environmental and neighborhood factors) achieved a 72.1% sensitivity rate, and a specificity rate of 67.8% (Table 3). High sensitivity corresponds to the proportion of presence cases that were correctly predicted as presence, while high specificity stands for the proportion of no-presence cases that were correctly identified as no-presence. Therefore, the variable selection for model 3 appeared to have the highest ability to correctly predict *A*. *albopictus* presence with a modest increases of 0.6% compared to model 1.

### Spatial patterns

**Fig 7:**
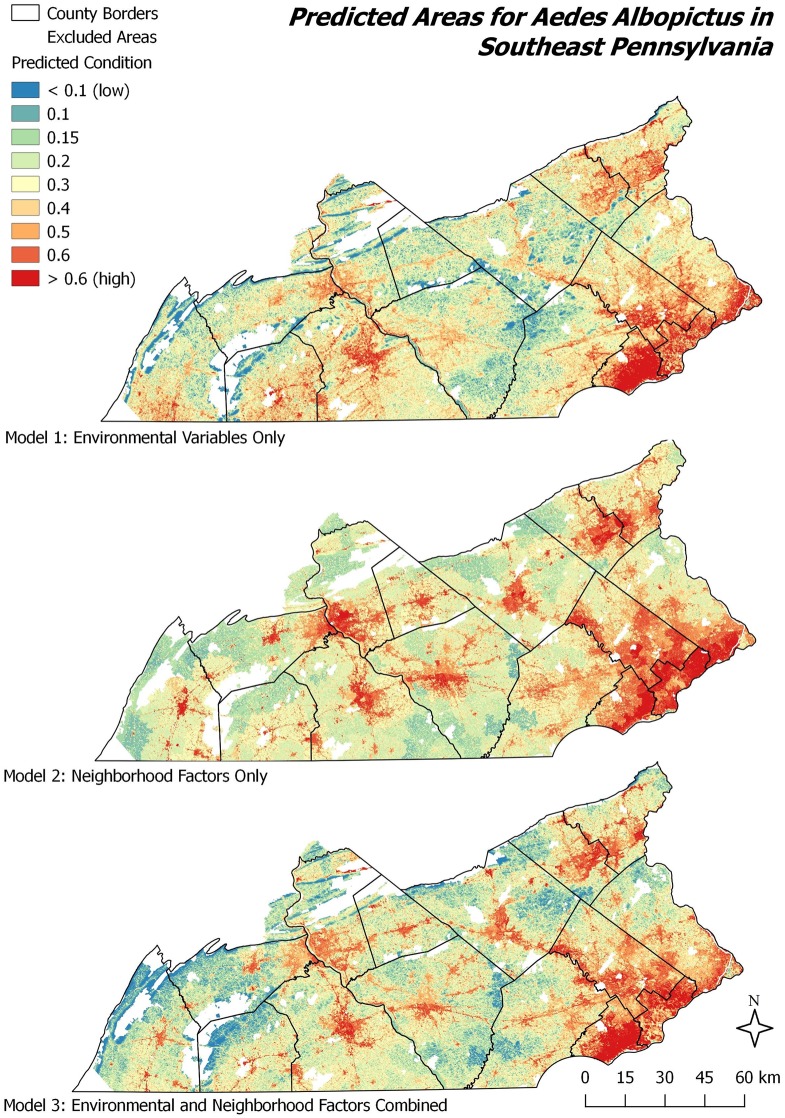
Habitat suitability for *A*. *albopictus* in the range from 0 to 1 (Logarithmic Output from MaxEnt 3.4.1).

**Fig 8:**
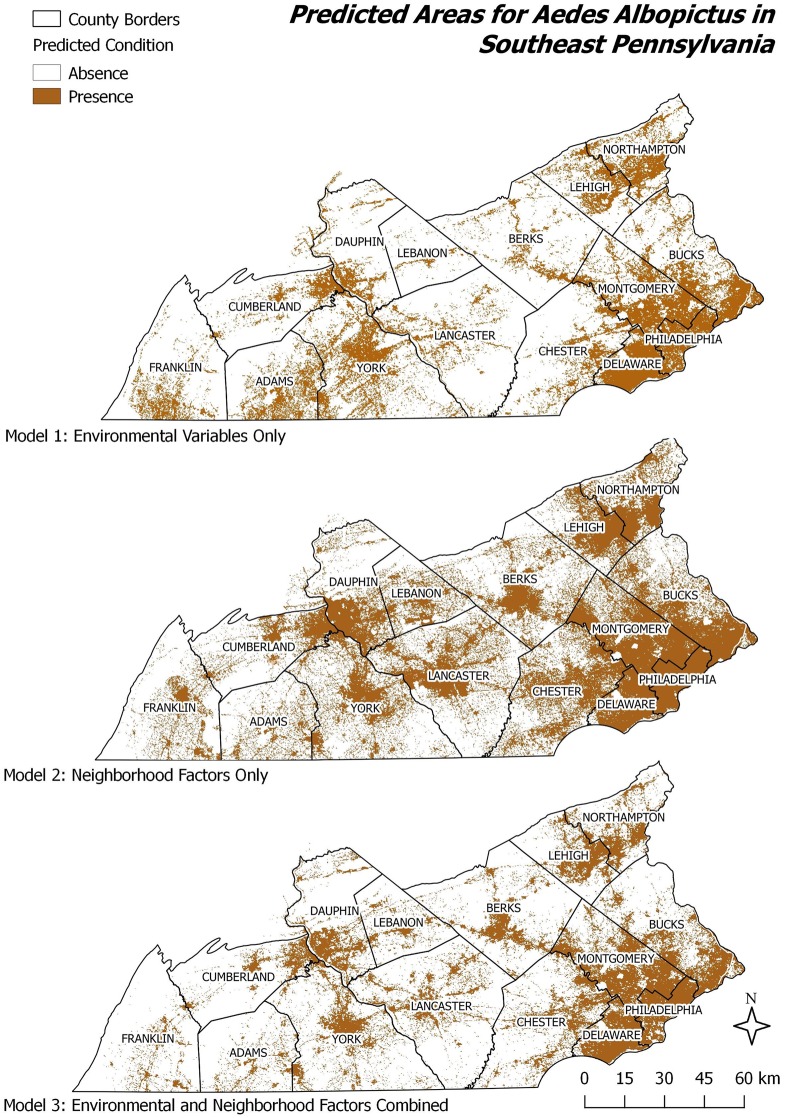
Habitat suitability for *A*. *albopictus* reclassified as presence/absence maps using MaxEnt’s thresholds based on maximum training sensitivity plus specificity occurrence method.

While comparing a models’ accuracy, sensitivity and specificity rates do not allow one to observe any major differences between model 1 (environmental) and model 3 (environmental and neighborhood factors) Each model type produced contrasting spatial patterns of predicted *A*. *albopictus* presence (Figs 7 and 8). While the environmental model 1 (Figs 7 & 8, upper maps) showed a higher suitability in rural and suburban regions along the Delaware River in the East, the neighborhood factors model 2 predicted the presence of *A*. *albopictus* in urban and urbanized centers as well as along major roads almost perfectly reflecting settlement patterns (Figs 7 and 8, middle maps). The combined model 3 (Figs 7 and 8, lower maps) predicted *A*. *albopictus*’ suitability in both urban and rural areas throughout the study region. Importantly, in the combined model 3, the presence of mosquitoes in rural areas is more constrained in comparison to model 1, while prediction conditions within urban areas were not as strong as in model 2. Some predicted patterns (e.g. Appalachian Piedmont areas in the West) look similar in model 2 compared to model 1 (environmental model), while others (Northern and Central urban areas) change based on neighborhood factors. All three models predicted the presence of *A*. *albopictus* in urban centers, however to different spatial extents. Model 1 covered largely Western regions, particularly in York, Adams and southern Franklin counties; this was in contrast to model 2, which predicted the presence in densely populated, urban settings. Model 3 predicted the *A*. *albopictus*’ presence in urban and urbanized places but not in remote, rural areas. However, unsuitable areas are found within urban places reflecting a low degree of presence conditions due to environmental constraints. Predicted mosquito locations under model 3 corresponded roughly to the intersection of the areas predicted by the environmental and neighborhood factors models 1 and 2. However, most urban centers captured by the neighborhood factors in model 2, are also predicted by model 3 (Figs 7 and 8).

## Discussion

### Summary of results

This study examined the predictive power of several environmental variables and neighborhood factors on *A*. *albopictus’* presence in SW Pennsylvania using MaxEnt version 3.4.1. Three models were compared using exclusively environmental variables (Model 1) or neighborhood factors (Model 2), and a model that combined the most influential variables from models one and two (Model 3). The results suggest that the combined model had a marginally higher ability to predict mosquito presence compared to other models. Moreover, the most influential variables in model 1 and model 2 appeared to be among the top-five most important variables in model 3. The strongest predictors included impervious surfaces, places of urban character (percent urban population >12), tree canopy (> 7%), EVI, as well as mild temperatures during the winter (ca. +2°C). It was also found that various types of land cover may influence *A*. *albopictus’* abundance differently and that the relationship between a predictive variable and *A*. *albopictus’* presence may have changed as new variables were added to the model.

### Summary of biological implications

The results from this study are similar to the findings of Vargas et al [48] and Paupy et al [49], who argue that warm temperatures and vegetation are typical for natural *A*. *albopictus’* habitat. Warm temperatures can, in general, positively influence the presence of *A*. *albopictus* during the coldest time of the year, assuming that mild winter conditions will secure species’ survival. Additionally, presence of vegetation cover was found to be an important predictor in the models. Similar to the spatial patterns in urban areas described by Honório et al [50], it was found that the presence of *A*. *albopictus* is expected in urban areas of SE Pennsylvania with sparse to high vegetation density. Adult mosquitoes feed on flower nectar [51], therefore the types of plants in the local environment could play a role on how “vegetation” is used by an *Aedes* species locally.

In addition to climatic conditions that determine *A*. *albopictus’* presence, humans are an important source of blood for these species [49], and therefore population density can contribute to the expansion of this species into new urban habitats. Therefore, variables that are direct indicators for human presence such as high population and housing density were important predictors. The role of impervious surfaces as a predictor of mosquito abundance can be explained in a few ways. First, roads and other paved areas are responsible for natural habitat fragmentation that was found essential for mosquito presence by Rochlin et al [25]. Second, even though the utilized impervious surface raster does not provide any information about road conditions, it could be assumed that high proportions of paved surfaces may increase the possibility of insufficient street conditions such as potholes, which may become puddles and potential mosquito breeding sites. However, in contrast to a study conducted by Little et al [27] in Baltimore, MD, a strong relationship was not found between mosquito presence and high proportion of vacant housing. This could be the result of differences in the scale of analysis (census block versus ZCTA) or an indicator that areas with lower population density are less attractive for *A*. *albopictus*. Among the most influential land cover classes, a strong positive association was found with woody wetlands, and a weaker positive association with mixed forest and developed lands. While negative associations were found with open water, bare soil, shrubs, pastures and croplands. Similar to other studies on land cover importance [22, 23, 25], the nature of land cover was found to be essential for *A*. *albopictus*’ presence.

Additionally, the results suggest that solely environmental factors are important but may be insufficient in predicting the presence of *A*. *albopictus* in highly urbanized, anthropogenic landscapes such as those of SE Pennsylvania. The inclusion of neighborhood factors can contribute to our understanding of spatial patterns of *A*. *albopictus*’ presence. Though the combination of both environmental and neighborhood factors did not essentially increase the accuracy in the prediction of mosquito frequency, the combined model 3 showed differences in the spatial patterns of mosquito distribution compared to the environmental or neighborhood factor models 1 and 2 alone.

The results from the combined model 3 suggests that the presence of *A*. *albopictus* is explained by a complexity of variables that go beyond those typically used for species-distribution models such as climate, vegetation or the degree of urbanization exclusively. The positive response of *A*. *albopictus* to impervious surfaces and urban population suggests that urban areas are becoming the main habitat for this mosquito. Future work could focus on assessing whether the importance of impervious surfaces on predicting mosquito presence is indirect due to a high predominance of imperviousness in urban and suburban areas or direct because water-impermeable surfaces offer breeding conditions alternative to artificial containers. Neighborhood measures can inform sustainable management decisions and also provided additional local information that can be used in possible interventions where behavioral modification is warranted (e.g. reduce standing water, remove trash, maintaining abandoned properties and streets).

### Summary of MaxEnt implications using both environment and neighborhood data

This research shows that MaxEnt is suitable for utilizing environmental and neighborhood condition variables in species-distribution modeling. However, interpretation of results might be complicated, while associations may change depending on the inclusion of predictive variables. According to Kuemmerle et al [52], MaxEnt is not sensitive to the collinearity of variables but may complicate the interpretation of response curves especially if variable are interrelated.

## Conclusion

While previous studies considered changes in climate and natural environment as primary causes for habitat expansion of vector species such *A*. *albopictus*, our results showed that neighborhood factors are also important predictors. With further exploration of these associations, more effective mosquito prevention strategies could be developed, especially for urban environments.

Our results showed that neighborhood factors are important and align with the findings of the previous study by Rochlin et al [27], who found a link between urbanization degree and human risk for West Nile Virus (WNV) (also transmitted by *A*. *albopictus*). Harrigan et al [53] and Brown et al [54] reported similar associations between WNV and neighborhood condition variables.

The incorporation of neighborhood factors into the models presented herein help to account for the impact of humans and the built environment, that was found to be influential by others [22, 23, 25, 27]. Finally, the prediction maps (Figs 7 & 8) in comparison to the urban characteristic of the study area (Fig 1) provide an opportunity to observe the previously mentioned “patterns of urbanization” of *A*. *albopictus*. Many areas predicted in the model 1 would fall outside the urban and urbanized areas, and were excluded in model 3. Analogously, several neighborhood condition factors from model 2 become less-influential contributors in model 3. Similar to previous findings, several environmental variables were found among the most influential factors. Nevertheless, our results indicate that the plasticity of *A*. *albopictus* allows it to exploit new environments.

Even though all models have statistical similarities, the difference in spatial patterns shows that some models might be more informative. Particularly, climate and natural environment are the essential requirements for mosquito presence, and must be included into any species distribution modeling. Therefore, model 1 would be sufficient especially for analyzing unpopulated places, where the interest is on exploration of the range distribution considering changes in climate or vegetation. However, in urban places, human factors are essential elements, because human activities shape and modify the environmental conditions and land cover structures that are known to influence the ability of mosquitoes to reproduce. Incorporating neighborhood factors provides for additional risk factors for mosquito presence because of higher human population density and anthropogenic modifications. Therefore, we regard model 3 to be the most informative when analyzing mosquito distribution in populated areas. Since mosquito management is targeted toward prevention and control of mosquitoes that spread viruses, model 3 provides additional information about mosquito distributions and therefore can better inform mosquito management, particularly for targeting appropriate actions for mitigating mosquito reproduction and subsequent public health interventions. Finally, model 2 with exclusively neighborhood factors is the least informative because it does not account for necessary climatic and environmental characteristics that influence the presence of this species. Therefore, we deem this model as insufficient to predict the presence of the species.

This study also shows that MaxEnt is suitable for species distribution modeling using area-based neighborhood factors of non-environmental origin. Consequently, further research incorporating historical demographic census data on various scales such as tracts or block groups could elucidate associations and trends between neighborhoods’ social environment/living conditions and presence of vector species. Moreover, while other machine learning methods may require advance programming knowledge, MaxEnt is relatively uncomplicated to operationalize and interpret.

Limitation in this study are mostly related to the effect of spatial bias and resampling of the input raster data on the predictive power of our models. Spatial bias is related to the uneven distribution of sampled mosquito locations in the landscape. We applied a spatial filter to mitigate the effect of spatial bias in the predictions. However, our results should be taken with caution considering the potential effects of any remaining bias in the filtered data. Developing a mosquito sampling strategy that would equally represent the chance of mosquito presence throughout the study area will likely improve the predictive power and reliability of the models substantially. An improved mosquito sampling scheme would also help better understanding differences in both accuracy and spatial predictions between models.

The effect of resampling has to do with the loss of information that occurs when the pixel size and geographic projection of input raster files with different spatial resolutions are aligned to the same reference resolution. Here the reference resolution was the 250m pixel size of the input MODIS data. This reference was considered a good compromise between the finer spatial resolution of the NLCD products (30m) and the coarser resolution of the PRISM climatic data (4 km). Resampling of NLCD products from 30 to 250 m results in data generalization. In our case, this generalization translates into the ability of the model to predict the presence of mosquitoes at a granularity no finer than 250m. Resampling of PRISM climate rasters from 4km to 250m will not capture local variations in climate within each 4km cell. We expect that the effect of the resampling of the climatic data will not affect our predictions substantially because weather in the study area is mostly affected by large regional climatic patterns rather than local orographic effects, especially considering the flat topography of the region.

Finally, further research could focus on exploring spatial differences in the ability of both neighborhood conditions and environmental variables to predict mosquito presence in urban and rural areas. Also, the influence of both environmental and neighborhood condition variables in the prediction of temporal variations in mosquito populations requires further exploration.

## Supporting information

S1 Table Coordinates of the remaining mosquito-presence locations. (CSV)

S2 Table Summary of applied MaxEnt (version 3.4.1) settings. (DOCX)
