# Supplementary material for: Integrating environmental and neighborhood factors in MaxEnt modeling to predict species distributions: A case study of Aedes albopictus in southeastern Pennsylvania
Source: PLoS One. 2019 Oct 17;14(10):e0223821. doi: 10.1371/journal.pone.0223821 (PMC6797167; doi:10.1371/journal.pone.0223821)
Supplement: S2 Table — (DOCX) [file pone.0223821.s002.docx]

**Supporting information**

**S2 Table: Summary of applied MaxEnt (version 3.4.1) settings**

| **Requirements** | **Entered Value** |
| --- | --- |
| Create response curves | Yes |
| Make picture of predictions | Yes |
| Do Jackknife to measure variable importance | Yes |
| Output format | Logistic |
| Output file type | asc |
| **Basic Settings**  **(if not listed, then used by default)** | **Entered Value** |
| Random test percentage | 30 |
| Maximum number of background points | 10000 |
| Replicated run type | Crossvalidate |
| **Advanced Settings**  **(if not listed, then used by default)** | **Entered Value** |
| Append summary results to maxentResults.csv file | Yes |
| Maximum iterations | 12000 |
| Adjust sample radium | 250 |
| **Experimental Settings**  **(if not listed, then used by default)** | **Entered Value** |
| Show exponent in response curves | Yes |
